# Factors influencing poor response to type 2 targeted therapies in severe asthma: a retrospective cohort study

**DOI:** 10.1186/s12890-023-02786-w

**Published:** 2023-12-05

**Authors:** Mona Al-Ahmad, Asmaa Ali, Ahmed Maher

**Affiliations:** 1 Microbiology Department, College of Medicine, Kuwait University, P.O. Box 24923, Kuwait City, 13110 Kuwait; 2 Department of Laboratory medicine, School of Medicine, Jiangsu University, Zhenjiang, 212013 P. R. China; 3 Department of Allergy, Al-Rashed allergy center, Ministry of Health, Kuwait City, Kuwait; 4 Department of Pulmonary Medicine, Abbassia Chest Hospital, MOH, Cairo, Egypt

**Keywords:** Severe asthma, Omalizumab, Benralizumab, Poor response

## Abstract

**Background:**

A significant breakthrough has been made in treating severe asthma, with the recognition of various asthma phenotypes and an updated management guideline. Type 2 targeted therapies, such as benralizumab and omalizumab; have been identified as an effective treatment for severe asthma, improving patient response, lung function tests and asthma symptom control. This study aimed to evaluate factors contributing to poor response to therapy.

**Methods:**

A retrospective single-center cohort study of 162 patients with severe asthma who started biologic therapy; their data were retrieved from medical records for further analysis. Poor responders were patients remained clinically and functionally uncontrolled despite even after augmenting all treatment options.

**Results:**

Childhood-onset asthma, bronchiectasis, poor symptom control (ACT below 19), severe airway obstruction (< 60% predicted), and maintenance oral corticosteroid (mOCS) use were significantly associated with poor response to omalizumab and benralizumab; *p* = 0.0.4 and 0.01; 0.003 and 0.01; 0.01 and 0.001, 0.05 and 0.04; 0.006 and 0.02, respectively. However, chronic rhinosinusitis and IgE < 220kIU/L were associated with higher poor response rates to omalizumab (*p* = 0.01 and 0.04, respectively). At the same time, female patients and those with blood eosinophils level < 500 cells/mm3 had a higher poor response rate to benralizumab (*p* = 0.02 and 0.01, respectively). Ischemic heart disease (IHD), bronchiectasis, and continued use of OCS increased the likelihood of poor response to omalizumab by 21, 7, and 24 times (*p* = 0.004, 0.008, and 0.004, respectively). In contrast, the female gender, childhood-onset asthma and higher BMI increased the likelihood of poor response to benralizumab by 7, 7 and 2 times more, *p* = 0.03, 0.02 and 0.05, respectively.

**Conclusion:**

Poor response to omalizumab treatment was independently associated with ischemic heart disease (IHD), bronchiectasis, and a history of maintenance oral corticosteroid (mOCS) use. Conversely, poor response to benralizumab therapy was independently linked to female gender, childhood-onset asthma and higher body mass index (BMI).

## Introduction

Asthma is a commonrespiratory disease that affects a large number ofpeople and can significantly impact their quality of life. It ranks 16th and 28th globally among the top cause of morbidity and mortality. Approximately 300 million people worldwide have asthma, which is expected to affect an additional 100 million by 2025 [1]. The prevalence, severity, and mortality rates of asthma vary across different regions, with higher rates in high-income countries but more deaths in low- and middle-income countries [2].

The concept of defining asthma severity as regarding the degree of airway obstruction was changed [3]. The patient was considered to have severe asthma if he did not respond well to current medication, which included systemic steroid [4–6]. The average rate of severe asthma was 5–10% of all asthmatic patients [5], and the idea behind the new definition of severe asthma depends on the underling pathogenesis [6].

In severe asthma, innate and adaptive immune system interplay together and conduct a cascade of airway hyper-reactivity, activation of epithelial cells with mucous over-production, and ended by airway remodeling. Different asthma phenotypes have emerged due to asthma pathogenesis heterogeneity, which describes the visible clinical variability; however, other different endotypes have been recognized at the molecular mechanism level [7].

Type II asthma is a condition that affects the lower airway with type II inflammation. It often involves eosinophilia and may or may not be related to allergies. The endotype of type II asthma includes T helper type 2 (Th2) cells, type 2 innate lymphoid cells (ILC2), IgE-secreting B cells, and eosinophils. Eosinophilic non-allergic asthma is primarily caused by ILC2, which produces IL-5 to recruit eosinophils into the airway lining. The other significant subgroup of asthma is non-type two asthma; a diverse group of endotypes and phenotypes, including exercise-induced, obesity-induced asthma and others [7–9].

Steroid therapy is the cardinal medication for type II inflammation in asthma, either through inhalation or oral administration. A significant reduction in FeNO and blood eosinophil counts is a positive indicator of medication effectiveness [9]. However, some patients with severe asthma may continue to experience airway eosinophilia despite high-dose inhaled or oral corticosteroids (ICS-OCS) [6].

New biologic therapies have been described as targeted therapy in severe asthma patients who do not respond to conventional medication, such as anti-IgE (Omalizumab) and anti-IL-5 (Benralizumab) (9–10). In 2009, omalizumab was approved as an add-on therapy for severe asthma. It works by attaching to the IgE in the blood, which can help reduce the frequency of asthma attacks and the need for OCS [11]. Benralizumab; A newer monoclonal therapy, approved in 2016, targets the IL-5 receptors on eosinophilic cells [12].

This study aimed to identify factors that contribute to poor asthma control, even with the addition of omalizumab or benralizumab to standard treatment medication. The study analyzed data from a real-world severe asthma registry in Kuwait.

## Methods

### Patients and study design

A retrospective study of a cohort of adult patients aged 18 years and above with severe asthma was conducted. These individuals underwent a comprehensive confirmation process to establish the diagnosis of asthma including medical history and clinical evaluation. Lung function was assessed using appropriate spirometry software, complemented by either a reversibility test or airway hyperreactivity test, tailored to each patient’s unique clinical presentation. Furthermore, comprehensive allergic and inflammatory markers were performed, including skin prick tests (SPT) to common aeroallergens (Diater, Spain), total serum immunoglobulin E (IgE) levels, blood eosinophil counts, and fractional exhaled nitric oxide (FeNO) levels.

Following a confirmation of severe asthma, asthma control was evaluated asthma control test (ACT) questionnaire and a careful tally of exacerbation events experienced by the patients over the preceding year. Additionally, the count of exacerbations necessitating hospitalization or admission to an intensive care unit (ICU) was recorded. These assessments were augmented by an analysis of the prescribed dosage of inhaled corticosteroids (ICS) in conjunction with any additional controller medications, and a record of oral corticosteroids (OCS) therapy usage spanning at least six months.

The definitive diagnosis of severe asthma was established following the criteria stipulated by the European Respiratory Society/American Thoracic Society [13]. The study cohort comprised individuals prescribed omalizumab or benralizumab as an adjunctive therapy between January 2010 and May 2023 at the Al-Rashed Allergy Center in Kuwait.

Omalizumab, in this study, is prescribed for patients who meet all the following criteria: Patients with severe persistent asthma, aged 18 years and above, with a documented positive skin test results or in vitro reactivity to perennial aeroallergens and experience frequent daytime symptoms or nighttime awakenings due to asthma, a history of asthma exacerbations that required systemic glucocorticoids despite being on a daily high-dose of ICS or a leukotriene receptor antagonist (LTRA) in combination with a long-acting beta-agonist (LABA), the total serum IgE levels within 30 to 1500 IU/mL, body weight should be between 20 and 150 kg, and the forced expiratory volume in 1 s (FEV1) should be < 80% (14–15). According to the algorithm for treating severe allergic asthma (SAA), omalizumab is the preferred medication after a thorough evaluation and elimination of other potential causes for high IgE levels above 1500 IU [16]. On the other hand, benralizumab eligibility criteria includes the following: patients with severe persistent asthma, aged 18 years and above, with blood eosinophil count ≥ 300/µL. Additionally, patients must have a history of asthma exacerbations that required systemic glucocorticoids despite being on a daily high-dose ofICS or a leukotriene receptor antagonist (LTRA) in combination with a long-acting beta-agonist (LABA). They should also have experienced at least two exacerbations in the previous 12 months or one asthma exacerbation in the last 12 months with chronic OCS [17].

Complete medical records were retrieved from all patients meeting the predefined inclusion criteria. Patients with incomplete or insufficient data records, those whoprematurely discontinued their biologic therapies and individuals receiving biologic treatments for less than one year prior to the study’s commencement were excluded from the analysis.

### Data collection and endpoints

The demographic and clinical data of patients were collected from the medical records in an Excel sheet; the data included basic demographic characteristics such as age, gender, smoking status, BMI, comorbidities, ACT score, FEV1%, FeNO, total serum IgE, and blood and sputum eosinophil levels.

Biological treatment satisfactory response (SR) was defined as meeting the following criteria: a reduction in asthma exacerbations of 50% or more compared to baseline, a 50% or more reduction in courses of oral corticosteroids (OCS) compared to baseline, meaningful improvement in ACT score of three or more from baseline, and a mean change of post-bronchodilator FEV1% of 150 ml or more compared to baseline [18], however, poor response (PR) is referred to patients who were still uncontrolledclinically and functionally even after augmenting all treatment options.

### Ethics approval and consent to participate

The study was approved by the Ethics committee of Kuwait University and the Ministry of Health (Research study number 2121/2022). All methods were carried out in accordance with relevant guidelines and regulations. Informed consent has been obtained from all participants involved in the study, as well as their legal guardians, to ensure that they are fully aware of the nature and purpose of the research, and have given their voluntary and informed consent to participate.

### Statistical analysis

We used an Excel sheet to collect and code the data and performed statistical analysis using SigmaPlot for Windows version 12.5 (Systat Software, Inc, UK, 2011). We represented mean and standard deviation (SD) for numerical data, while categorical data was represented in the form of numbers and percentages. We used independent t-tests, Mann-Whitney tests to compare two means or medians, and chi-square tests for frequency comparison. We performed logistic regression analysis using a backward elimination technique to identify potential predictors of poor response to omalizumab and benralizumab. All tests were two-sided, and we considered a p-value less than 0.05 to be significant.

## Results

### Characters of patients with severe asthma

In Tables 1 and 162 patients were diagnosed with severe asthma and eligible for biological therapy. The mean age was 45 [13] years, and the mean BMI was 32 kg/m^2^. 64.81% of cases were female, 21.6% were current smokers, and 43.21% had Childhood-onset asthma. Respiratory system-related comorbidities were the most frequent; allergic rhinitis 55.56%, chronic rhino sinusitis 48.77%, nasal polyposis 26.54%, and bronchiectasis 19.75%.

**Table 1:** Patient’s Characteristics

Factors	Total (n = 162)	
	Mean	SD
Age	44.95	12.84
BMI	32.07	8.22
	N	%
F-sex	105	64.81
Current smoker	35	21.60
Childhood onset asthma	70	43.21
Comorbidity	N	%
DM	31	19.14
HTN	32	19.75
IHD	15	9.26
Obesity	90	55.56
GERD	24	14.82
AR	90	55.56
CRS	79	48.77
NP	43	26.54
Bronchiectasis	32	19.75
Eczema	9	5.55
Urticaria	18	11.11
	Mean	Range
ACT	13.25	(5–25)
FEV1%	58.25	(21–109)
Total IgE (KU/L)	523.9	(10-5000)
FeNO (PPB)	26.35	(1-119)
Blood Eosinophils	499.9	(1-3460)

Numerical data presented as mean and stander deviation, or range (minimum-maximum), and categorical data as number and percentage. N: number, SD: standard deviation,BMI: body mass index, min: minimum, max: maximum, ACT: asthma control test, FEV1: forced expiratory volume in one second, IgE: immune globulin E, FeNO: fractional exhaled nitric oxide, PPB: part per billion

Prior to biologic therapy, the work-up revealed a lack of symptom control (mean ACT 13), marked airway obstruction (mean FEV1%predicted 58.25%), intermediate to high levels of inflammation (mean total IgE 523.9uL/mL,max 5000uL/mL;mean FeNO26.35 ppb, max 119 ppb; mean blood eosinophilscount499.9 cells/microL, max 3460 cells/microL.

### Factors associated with poor response

In Tables 2 and 133 patients were treated with omalizumab and 29 with benralizumab. Childhood-onset asthma, bronchiectasis, poor symptom control (ACT below 19), severe airway obstruction (mean FEV1 predicted < 60%),and maintenance oral corticosteroid (mOCS) use were significantly associated with poor response to omalizumab and benralizumab; p = 0.0.4 and 0.01; 0.003 and 0.01; 0.01 and 0.001, 0.05 and 0.04; 0.006 and 0.02, respectively. However, chronic rhinosinusitisand IgE below 220kIU/L were associated with higher poor response rates to omalizumab (p = 0.01 and 0.04, respectively). At the same time, female patients and those with blood eosinophils count<500 cells/mm^3^ had a higher poor response rate to benralizumab (p = 0.02 and 0.01, respectively). In the omalizumab group, 66.67% of patients with poor response required a switch to an alternative biologic therapy, while 92.31% of patients in the benralizumab group needed to switch.

**Table 2:** Factors affecting poor response to Omalizumab or Benralizumab

Factors	Omalizumab (n = 133)					Benralizumab (n = 29)				
	Overall responder (n = 115)		Poor responder (n = 18)		P	Overall responder (n = 16)		Poor responder (n = 13)		P
Age	45	12.9	42.7	11.8	0.46	49.1	14.5	42.8	11.2	0.21
BMI	32.13	8.78	31.15	7.08	0.63	31.31	6.23	34.93	5.83	0.12
F-sex	75	65.22	12	66.67	0.91	7	43.75	11	84.62	**0.02**
Current smoker	23	20	5	27.78	0.45	4	25	3	23.08	0.91
Childhood onset asthma	47	40.87	12	66.67	**0.04**	3	18.75	8	61.54	**0.01**
Comorbidity										
DM	20	17.39	4	22.22	0.62	5	31.25	2	15.38	0.32
HTN	21	18.26	4	22.22	0.68	5	31.25	2	15.38	0.32
IHD	7	6.09	3	16.67	0.11	2	12.5	3	23.08	0.45
GERD	62	53.91	10	55.56	0.89	1	6.25	2	15.38	0.42
Obesity	18	15.65	3	16.67	0.91	11	68.75	7	53.85	0.41
AR	61	53.04	12	66.67	0.27	8	50	9	69.23	0.29
CRS	48	41.74	13	72.22	**0.01**	9	56.25	9	69.23	0.47
NP	26	22.61	5	27.78	0.63	7	43.75	5	38.46	0.77
Bronchiectasis	17	14.78	8	44.44	**0.003**	1	6.25	6	46.15	**0.01**
Eczema	4	3.48	1	5.56	0.52	1	6.25	2	15.38	0.42
Urticaria	10	8.7	2	11.11	0.66	0	0	1	7.69	0.44
ACT										
Less Than 19	96	84.21	18	100	**0.01**	8	50	13	100	**0.001**
More than 20	18	15.79	0	0		8	50	0	0	
FEV1%										
More than 60%	63	54.78	6	33.33	**0.05**	8	50	2	15.38	**0.04**
Less than 60%	52	45.22	12	66.67		8	50	11	84.62	
Total IgE (KU/L)										
Below 220 KIU/L	46	41.07	10	55.56	**0.04**	4	25	6	53.85	0.22
From 221–700 KIU/L	46	41.07	2	11.11		4	25	3	23.08	
More than 700 KIU/L	20	17.86	6	33.33		8	50	3	23.08	
Blood Eosinophils										
Less than 500 cells/mm^3^	25	43.86	6	46.15	0.88	2	12.5	7	53.85	**0.01**
More than 500 cells/mm^3^	32	56.14	7	53.85		14	87.5	6	46.15	
FeNO (PPB)										
Less than 25 (PPB)	28	66.67	2	50	0.51	5	31.25	6	46.15	0.52
More than 25 (PPB)	14	33.33	2	50		11	68.75	7	53.85	
Maintenance OCS	3	2.61	4	22.22	**0.006**	0	0	3	23.08	**0.02**
Treatment shift	12	10.43	12	66.67	**0.001**	10	62.5	12	92.31	**0.04**
Treatment duration	7.87	2.96	8.39	2.2	0.38	5.63	1.07	4.11	1.12	0.19

Numerical data represented as mean and standard deviation, and categorical data as number and percentage, N: number, SD: standard deviation,BMI: body mass index, AR: allergic rhinitis, CRS: chronic rhino sinusitis, NP: nasal polyposis, DM: diabetes mellitus, HTN: hypertention, IHD: ischemic heart disease, GERD: gastro esophageal reflux disease. †: Independent t-test, §: Chi square test, P < 0.05 considered significant

### Predictors of poor response

In Table 3, the probability of a poor response to omalizumab rose 21 and 7 times higher in patients with IHD and bronchiectasis (p = 0.004 and 0.008, respectively). Furthermore, continued use of OCS increased the likelihood of poor response by 24 times (p = 0.004). However, the factors predicting a poor response to benralizumab were distinct. The female gender and childhood-onset asthmaraised the probability of a poor response 7 times (p = 0.03 and 0.02, respectively); additionally, for every 1 unit increase in BMI, the likelihood of a poor response to benralizumab increased by two times (p = 0.05).

**Table 3:** Predictors of poor response toward omalizumab and benralizumab

Omalizumab			
Factors	OR	95% CI	P
Age	0.98	(0.9279,1.0327)	0.434
BMI	0.99	(0.9101,1.0703)	0.743
F-Sex	2.40	(0.5750,10.0029)	0.208
IHD	21.24	(2.7042,166.7854)	**0.004**
Bronchiectasis	7.02	(1.6755,29.3819)	**0.008**
Maintenance OCS	23.71	(2.8799,195.1335)	**0.004**
**Benralizumab**			
**Factors**	**OR**	**95% CI**	**P**
Age	0.98	(0.9047,1.0686)	0.68
BMI	1.64	(0.9507,1.6530)	**0.05**
F-Sex	7.32	(0.9900,54.2544)	**0.03**
Childhood onset asthma	7.17	(1.0984,46.8617)	**0.02**
FEV1% (< 60%)	2.94	(0.2621,33.1945)	0.36

Logistic regression model with backward elimination, Goodness of fit test: Hosmer-Lemeshow, X^2^ = 11.16, P = 0.19, Coef: coefficient, OR: odd ratio, CI: confidence interval,P < 0.05 considered significant

## Discussion

The present study analyzed factors associated with poor response to omalizumab and benralizumab, biologic therapy used in severe asthma as add-on therapy. Our patients with asthma have a high prevalence of allergic rhinitis and chronic rhinosinusitis as respiratory diseases. The second most common comorbidity is systemic diseases (HTN and DM). However, eczema and urticaria are the least associated comorbidities. Another study found a higher incidence of comorbidities such as rhinitis, sinusitis, and GERD [19]. In the same line, a meta-analysis included 11 studies with a total of 117,548 asthma patients in comparison with 443,948 non-asthma controls revealed that the presence of cardiovascular diseases, cerebrovascular diseases, hypertension, obesity, and respiratory comorbidities was among the most predominant comorbid diseases in asthmatic patients [20]. Similar results were concluded in Novelli et al. study [21].

Several studies have evaluated omalizumab effectiveness in treating severe asthma; a randomized controlled trial discovered that severe asthma patients with high blood eosinophil counts, FeNO levels, and serum periostin experienced a significant reduction in exacerbation frequency [22]. However, eosinophilic rhinosinusitis is associated with treatment failure with omalizumab, and received benralizumab as an alternative therapy [23]. That came in concordance with our data; CRS was associated with poor response to omalizumab, in addition, all cases on benralizumab had been switched from omalizumab.

The prevalence of asthma with CRS ranges from 22 to 42% (24–25), increasing to 45% in severe asthma patients [26]. These findings provide evidence for a two-way connection between CRS and asthma severity, supporting the unified airway theory. Furthermore, CRS with nasal polyp share similar pathogenesis of severe asthma, it characterized by type 2 inflammation with excess esinophils infiltration and IgE level [27].

In real-world data, omalizumab found to be effective in severe allergic asthma, including those with CRSwNP [28]. Conversely, there is limited real-life evidence about the clinical profile of patients who respond to benralizumab after ineffective mepolizumab treatment [29]. A large study reported the effectiveness of benralizumab in patients who did not respond to mepolizumab or reslizumab. Nevertheless, the study did not include a subgroup-focused analysis to eliminate potential confounding factors related to reslizumab dosage [30].Hamada et al. [31] found that patients with chronic eosinophilic rhinosinusitis responded better to benralizumab than mepolizumab, consistent with our findings. However, their report did not provide details on when to switch or identify predictors of better response to benralizumab. We observed that CRS did not correlate with poor response to benralizumab, but rather with omalizumab; 72.22% of poor response group had CRS.

The present study found that the associated bronchiectasis was significantly linked to omalizumab and benralizumab’s poor response. Distinguishing between asthma and bronchiectasis can be challenging due to shared symptoms and physiological similarities; the two conditions have significant overlap, with varying comorbidity rates. Roughly 20% of bronchiectasis patients have eosinophilic inflammation, 7–46% have comorbid asthma, and severe asthma is limited to 3.3%. Type-2-targeted biologics have shown efficacy in treating eosinophilic bronchiectasis and potentially other cases before oral corticosteroids [32]. However, recognizing and addressing comorbidities associated with asthma, such as bronchiectasis, is crucial to prevent treatment failure, exacerbation of symptoms, and increased healthcare costs. Bronchiectasis and asthma often coexist, exacerbating airway inflammation and asthma symptoms [32]. Although their interaction has not been extensively studied, common traits such as mucus plugging, infections, and inflammation suggest a potential link [33].In our study, severe asthma patients with bronchiectasis were those who developed bronchiectasis secondary to severe asthma, and not at the initial asthma diagnosis, were included.

High-resolution CT scans are a reliable method to confirm bronchiectasis while using the NOPES score (FeNO, Pneumonia, Exacerbations, and Severity) helps assess risk [33–36]. It is important to personalize treatment strategies for each disease and use precise therapy for asthma (37–38). ICS may be suitable for asthma and COPD, but they are controversial for bronchiectasis [39].For patients with steroid-dependent severe asthma and bronchiectasis, various therapies are available to address each condition [33]. These treatments can include options for airway clearance, pulmonary rehabilitation; long-term antibiotics, anti-inflammatory medicines, and standard asthma care (32–33). Moreover, it should be noted that eosinophilic inflammation is the root cause of asthma and can be managed with corticosteroids [40]. Conversely, bronchiectasis is mainly neutrophilic, and macrolides have been found to reduce exacerbations [41]. In some cases, patients who do not respond to asthma treatment may have an underlying bronchiectasis [32], and macrolides can be used [33].

The AMAZES study demonstrates the importance of both short- and long-term macrolide treatments for treating bronchiectasis in patients with asthma [42]. Additionally, biologic therapies like mepolizumab can assist in controlling severe asthma and may benefit those with bronchiectasis [43]. Nevertheless, further research is necessary to investigate the role of eosinophils and the effectiveness of anti-eosinophilic treatments in this context.

On the other hopeful side, patients with bronchiectasis that responded well to omalizumab could refer to the presence of associated Allergic bronchopulmonary aspergillosis (ABPA). Hence, out of 25 patients with bronchiectasis, 7 had ABPA, and all seven responded well to omalizumab. ABPA is a disease that arises when the lungs overreact to Aspergillus spp.; typically, it affects individuals with atopic asthma and cystic fibrosis, and A. fumigatus is the leading cause [44]. ABPA is prevalent in asthmatic patients with a range of 1.0–3.5%, but it can increase to 7–28% in individuals with corticosteroid-dependent asthma (45–46).

Aspergillus-related diseases can lead to bronchiectasis development, as seen in ABPA. They can also complicate existing bronchiectasis, such as aspergillomas in post-tuberculosis bronchiectasis [44]. Additionally, primary conditions like COPD can independently raise the risk of Aspergillus-related diseases and bronchiectasis (44–45).Omalizumab showed promise in reducing exacerbations, hospitalizations, and corticosteroid use in ABPA patients, improving their quality of life (47–48). However, limited studies explore its use in non-cystic fibrosis asthma patients with ABPA, leading to controversy (47, 49–50). Omalizumab reduced the need for corticosteroids, enhanced asthma management, and even permitted patients to discontinue systemic corticosteroids [46]. Research has suggested it may be a beneficial supplementary treatment for patients with refractory ABPA (47–48). Nonetheless, further investigation is required to establish its exact function, recommended dosage, and treatment period for these individuals.

We found that the female patients, higher BMI and childhood onset asthmaincreased the likelihood of poor response to benralizumab but not omalizumab. In contrast, continued use of OCS increased the likelihood of poor response to omalizumab 24 fold.

Female sex hormones significantly impact asthma control throughout a person’s life [51]. After puberty, women are more likely to have asthma than men(52–53). Additionally, obesity is strongly linked to asthma in women, which can sometimes be confused with obesity-related breathing difficulties (54–55). Women with both obesity and asthma have a unique type of asthma called neutrophilic asthma(56–57), which has a higher morbidity rate and a poorer response to inhaled corticosteroids [58]. Testosterone and oral contraceptive use are negative predictors of sputum neutrophils in this phenotype(59–60). In addition, neutrophilic asthma is associated with corticosteroid insensitivity, and neutrophil activation in the airway is widely reported in this phenotype. Otherwise, continues use of oral steroid may influence the delay apoptosis of neutrophilis, altered the pulmonary microbiome and facilitate chronic infection [61]. Furthermore, there is no difference between genders in asthma medication prescription patterns, except for the more frequent use of oral corticosteroids in women(62–63). However, to date, there are no studies on gender differences in prescribing biologics. Therefore, gender bias in clinical trials of mabs in treating severe bronchial asthma is a concern; female is more presented. Nevertheless, men may receive more aggressive treatments despite women reporting worse symptoms than men, and the underrepresentation of men in trials may lead to biased outcomes(64–65).

The poor response of omalizumab or benralizumab in controlling asthma symptoms may be due to several factors, such as neutrophilic overlap endotype, different types of airway inflammation, or resistance to corticosteroids. Additionally, non-inflammatory pathways like airway hyper-reactivity and remodeling may contribute to the issue.

On another side, the dose-related insufficient response may be explained the modest response of benralizumab in obese asthmatic patients, however, future real-world studies should be focus on the possibility of this hypothesis. Nevertheless, the acceptable elucidation for the poor response of benralizumab in patients with higher BMI could be related to the emergence of non-type two inflammations [66].

Research suggests that patients with high BMI levels may experience reduced efficacy of benralizumab due to systemic inflammation caused by elevated levels of obesity-related cytokines like TNF-α, IL-6, and leptin [66]. Factors like smoking or non-type two inflammations can also hinder the drug’s effectiveness, primarily targeting amplified type 2 immunity.

A recent study revealed that patients with severe eosinophilic asthma with enhanced gene expression related to neutrophilic activity exhibited poor responses to benralizumab [67]. Another study found that comorbid obesity in these patients was linked to a more significant disease burden, making it harder to alleviate symptoms and exacerbations with benralizumab [68]. While it is unclear whether obesity-related inflammatory pathways perpetuate this residual burden, more research is needed to determine the role of these pathways in specific individuals.

Childhood-onset asthma can significantly impact an individual’s health, especially regarding the possibility of severe asthma. Our data showed that childhood-onset asthma significantly predicts poor response to benralizumab. Sadly, approximately 30% of children with asthma continue to have the condition into adulthood [69], which can increase the risk of poor asthma control over time. However, limited documentation exists regarding the characteristics of adults who have had asthma since childhood. A recent study involving 1443 patients found that the persistent asthma group was younger and had fewer smokers. Furthermore, the findings suggest that adult patients who have experienced asthma persisting from childhood into adulthood tend to exhibit poorer lung function and more severe asthma symptoms compared to those with adult-onset asthma. These results imply that asthma that persists from childhood to adulthood may represent a distinct clinical phenotype of adult asthma [69].

Based on our findings, patients who had poor symptom control (ACT below 19) and severe airway obstruction (< 60% predicted) had higher rates of poor response to both omalizumab and benralizumab. However, IgE levels below 220kIU/L were linked to omalizumab’s poor response; nevertheless, those with a blood eosinophil level below 500 (cells/mm3) had a higher rate of poor response to benralizumab.

While some studies have not found a significant increase in lung function (FEV1) with omalizumab [70–72], others have shown improvement over time in asthmatic patients [73–76]. One study even found sustained improvement nine years after treatment compared to baseline [77]. Our study found that patients with airway obstruction (FEV1 < 60% predicted) responded poorly to omalizumab and benralizumab, that came in contrast with a recent study, they reported that benralizumab was associated with a significant improvement in FEV1 after just six days of administration [78], as well as better outcomes for patients with high levels of eosinophilia and more exacerbations [79]. While results may vary between individuals, these findings suggest that benralizumab may be a more effective treatment option for some asthma patients.

Asthma is a multifaceted condition with various subtypes based on bronchodilator responsiveness (BDR) and fixed airflow obstruction (FAO). Recent research indicates that nearly half of all patients with severe asthma exhibit FAO [80]. The study also reveals that omalizumab is more effective than a placebo in reducing exacerbations, particularly in patients with high BDR, regardless of their FAO status. Notably, patients with both FAO and high BDR experienced significantly improved lung function after receiving omalizumab treatment. These results suggested that omalizumab can effectively target the inflammation and airway hyperresponsiveness associated with this specific asthma subtype [80].On the other hand, asthma with low BDR may prove to be a more challenging and less responsive phenotype to omalizumab [80]. However, a different study suggests that benralizumab shows promise as a therapeutic option for patients with severe, uncontrolled eosinophilic asthma, especially those with FAO [81]. Several factors may account for the discrepancy with the previous study, including that patients with poor responses to benralizumab had low eosinophils blood counts and were taking mOCS.

## Conclusion

Our study showed some predictors for poor response to biologics in patients with severe asthma, which include IHD, being on mOCS and bronchiectasis for omalizumab, female sex, being overweight, and childhood-onset asthma for benralizumab. So far, analyzing bronchial secretions through cytological or bacteriological culture techniques, including sputum induction and bronchoscopic lavage, is invaluable in managing asthmatic patients with suboptimal responses to biological therapy. While sputum samples are the initial preferred option for their non-intrusiveness, bronchoscopic sampling remains a valuable alternative in cases where definitive results are elusive. This comprehensive examination of bronchial secretions aids in personalizing treatment strategies by identifying the nature of inflammation, distinguishing non-type 2 inflammation with the presence of neutrophils and other inflammatory cells, detecting potential infections that may mimic asthma symptoms, assessing treatment compliance through medication residue analysis, and providing insights into airway remodeling. Incorporating these insights into clinical practice can significantly enhance our ability to tailor therapies and improve outcomes for patients facing challenges with biological therapy responsiveness.

## Data Availability

The datasets used during the current study available from the corresponding author on reasonable request.
